# Prediction of Protein Acetylation Sites using Kernel Naive Bayes Classifier Based on Protein Sequences Profiling

**DOI:** 10.6026/97320630014213

**Published:** 2018-05-31

**Authors:** Md. Shakil Ahmed, Md. Shahjaman, Enamul Kabir, Md. Kamruzzaman

**Affiliations:** 1 Department of Statistics, University of Rajshahi, Rajshahi-6205, Bangladesh; 2 Department of Statistics, Begum Rokeya University, Rangpur-5400, Bangladesh; 3 School of Agricultural, Computational and Environmental Sciences, University of Southern Queensland, Australia; 4 Data Science for Knowledge Creation Research Center, Seoul National University, Korea

**Keywords:** Acetylation, Protein Sequences, Kernel Naive Bayes Classifier, Binary Encoding, CKSAAP Encoding, Kruskal-Wallis test

## Abstract

Lysine acetylation is one of the decisive categories of protein post-translational modification (PTM), it is convoluted in many significant cellular developments and severe diseases in the biological system. The experimental identification of protein-acetylated sites is painstaking, time-consuming and expensive. Hence, there is significant interest in the development of computational approaches for consistent prediction of acetylation sites using protein sequences. Features selection from protein sequences plays a significant role for acetylation sites prediction. We describe an improved feature selection approach for acetylation sites prediction based on kernel naive Bayes classifier (KNBC). We have shown that KNBC generated from selected features by a new feature selection method outperforms than the existing methods for identification of acetylation sites. The sensitivity, specificity, ACC (Accuracy), MCC (Matthews Correlation Coefficient) and AUC (Area under Curve of ROC) in our proposed method are as follows 80.71%, 93.39%, 76.73%, 41.37% and 83.0% with the optimum window size is 47. Thus the kernel naive Bayes classifier finds application in acetylation site prediction.

## Background

The lysine residues in a protein are acetylation for exist the acetyle group in the N terminus. The lysine acetylation is one of the most vital for a lot of cellular progressions [1, 2, 3, 4, 5]. For example, the dynamic interaction between lysine acetyl transferases (KATs) and lysine deacetylases (KDACs) is used to maintain the appropriate levels of histone acetylation for normal cell growth, proliferation and differentiation [6]. Acetylation has been shown to regulate of protein expression, complex steadiness, localization and fusion [7, 8, 9, 10, 11, 12]. Lysine acetylation is intricate in the thoughtful diseases comparable with the cancer for the abnormality of KAT/KDAC function of impacting the cell division [13, 14, 15]. The significant aims of the biological research are to describe the genome perspectives and recognize the function of genetic material in the post-genomic period [16]. For understanding the genome backgrounds the significant information can be provided by the proteomics and transcriptomics data [17, 18]. The acetylation is one of the most significant protein modifications with an important impact on the protein functions based on the proteomic data. In the amino acid it is frequently catalyzed through acetyl transferase that transmissions acetyl group of the acetyl coenzyme (Acetyl-CoA). A large scale of the mammalian acetylated proteins has been notorious via the proteomics techniques and which are suggesting that the acetylation may be as the ubiquitous as phosphorylation [6, 19]. The human proteins of 85% and yeast proteins of 68% were acetylated at N-terminus is described by Van Damme [20], The two forms of Acetylation occur in cellular methods such as Na-acetylation and Neacetylation. Na-acetylation is the irreversible modification happens during the translation of protein at N-terminus and the posttranslational practice it arises only for the chloroplast proteins [21, 22]. On the other hand the Ne-acetylation is the reversible post-translational modification and it can be happens in a protein at unfixed positions. Nevertheless, the apparatus of protein acetylation is the tranquil mostly unidentified. The first stage to apprehend the acetylation contrivance id to identify acetylation sites and certain diseases action it can be provided the certain guidance [23]. For the identification of acetylation sites the radioactivity detection [24], immunity affinity recognition, the chromatin immune precipitation (ChIP) [25] and mass spectrometric detection [26] are widely used experimental methods. All the early mentioned methods are the strenuous and time-intense. Exclusively, to classify a huge amount of acetylation loci are not capable quickly by the experimental methods. There are many computational methods are needed for prediction of acetylation sites from the large amount of acetylation datasets. Recently, there are several computational prototypes has been suggested for the acetylated lysine sites prediction in the literature [27, 28, 29, 30, 31, 32, 33]. Most of the existing computational methods are not so high accuracy and other performance evaluation methods rate to classify acetylation sites based on the protein sequences. Our proposed computational approach is most powerful and efficient for getting the better performance than the other existing computational methods.

## Methodology

### Datasets

The related datasets are collected from two-protein posttranslational modification sites (PTM) database, which is available in the online. The datasets are collected from the posttranslational modification (PTM) database SysPTM2 and another database is PhosphoSitePlus [34] for lysine acetylation sites prediction.

### Binary Encoding Scheme

The binary encoding scheme was carried out to covert sequence to numeric data matrix. To ensure the binary encoding with a unified length for every site can be characterized using the sequence fragments. We assigned a non-existing amino acid O (gap between two amino acid) to fill in the corresponding positions. Thus the natural alphabetic order of 21 different amino acids are reflected for the binary encoding, which are ordered as ACDEFGHIKLMNPQRSTVWYO.

### CKSAAP Encoding Scheme

In this study, acetylation and non-acetylation sites are predicted using the sequence encoding CKSAAP (Composition of K-spaced Amino Acid Pairs) method. By introducing an additional symbol "O" for a terminal space, CKSAAP is capable of investigating the incurable likings. In this study we used k = 0, 1, 2, 3, 4 to encode of acetylation and non-acetylation sites as the input feature vector.

### Feature Selection Method

In machine learning and statistics, feature selection, also known as variable or attribute selection or the variable subgroup selection is a procedure of selecting a subset of relevant features for use in model construction. In this study the feature data matrix is so high such as 12,000x26,000 dimensional, it is so difficult to classify without feature selection. We used the Kruskal-Wallis test statistics for feature selection because of our data matrix has no pattern like as normal distribution. It is a nonparametric method; the Kruskal-Wallis test doesn't follow the assumption of normality for residuals, different analogous it is also the one-way ANOVA. At first the data are ranking for all groups together according to their p-values; i.e. rank the data from 1 to N ignoring group membership. Assign for any tied values the average of ranks they would have received had they not been tied.

### Kernel Density Estimation

The conditional probability (XI |C = c) that the feature value in the ith position is of given class c, were estimated using KDE (Kernel Density Estimation) from a set of labeled training data (X, C). KDE is the methodology for estimating the probability density function of a given population by non-parametric manner [35].

### Naive Bayes Classifier

**Figure 1:**
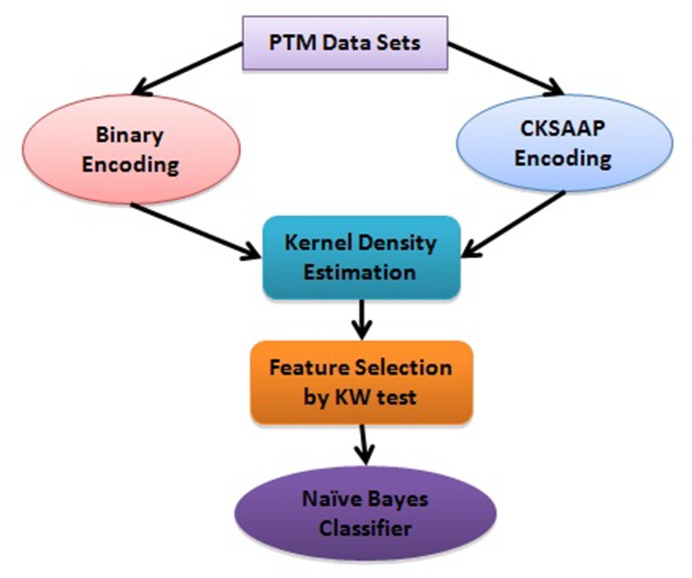
Schematic diagram for Kernel Naive Bayes modeling

The Naive Bayes Classifier (NBC) is generally known as a simple probabilistic classifier and assumes all the features are independent for a given class as shown in Figure 1. For greatly reduce the complexity of development of the classifier by this assumption. The input X = (x1, x2, ... xp) for the naive Bayes classifier produce a binary class C ∈{+1, -1}, where +1 denotes that the residue was predicted as in acetylation and -1 denotes the residues non-acetylation. The NB classifier was trained by a set of categorized training dataset (X, C). Then the residue of the input X was classified as +1 (acetylation) otherwise -1 (non-acetylation) and θ is the classification threshold. In our study the optimal threshold is θ = 0.37.

### Performance Assessment Methods

The following measures were calculated to assess the performance of kernel Naive Bayes classifier, using counts of true positives (TP; residues correctly predicted as acetylation), false positives (FP; residues incorrectly predicted as acetylation or Type-I Error), true negatives (TN; residues correctly predicted as non-acetylation) and false negatives (FN; residues incorrectly predicted as non-acetylation or Type-II Error).

ACC: Accuracy (ACC) is the proportion of the known residues that are correctly predicted in all prediction and is defined as ACC=(TP+TN)/(TP+FN+TN+FP)

MCC: Matthews Correlation Coefficient (MCC) indicates the degree of the correlation between the actual and predicted classes of the residues. MCC values range between -1 ≤ MCC ≤ +1, +1 means all the predictions are correct, and -1 means none of them are correct. The MCC can be defined as MCC= ((TPxTN)-(FPxFN))) √((TP+FP)x(TP+FN)x(TN+FP)x(TN+FN))

Sensitivity: The sensitivity that is True Positive Rate (TPR) measures the proportion of the known acetylation residues that are correctly predicted as acetylation residues. The sensitivity is defined as Sensitivity= TP/(TP+FP)

Specificity: The specificity or True Negative Rate (TNR) measures the proportion of the known non-acetylation residues that are correctly predicted as non-acetylation residues. The specificity can be defined as Specificity=TN/(TN+FP)

## Results & Discussion

**Figure 2:**
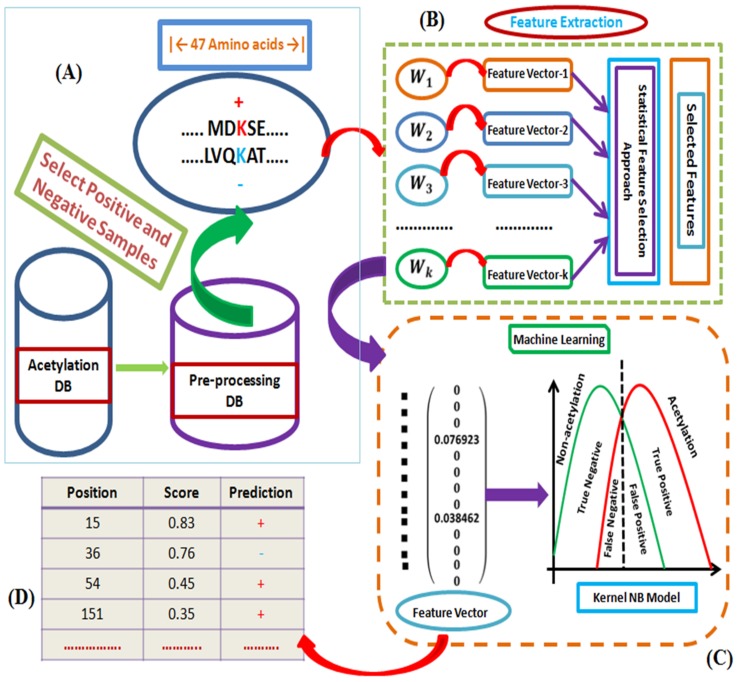
Flow chart for the prediction of acetylation and non-acetylation sites using kernel NB model based on the K-W feature selection (Proposed), the necessary working steps are as follows: (A) Data collection, preprocessing and making positive and negative groups using suitable window size. (B) Feature extraction from the fragment of protein sequences using Perl programming language. (C) Machine learning algorithm kernel naive Bayes for the classification of acetylation sites. (D) The prediction score and results by our proposed method.

For identification of acetylation and non-acetylation sites from protein sequences to make numerical data matrix using CKKSAAP and binary encoding scheme workflow shown in Figure 2. From Table 1 the AUC is 0.830 for CKSAAP encoding method and binary encoding method AUC is 0.761. Hence, the performance of the CKSAAP encoding is better than the binary encoding method. Though the feature selection method brought no significant performance to improved method that can be needed to find out the most important features (amino acid pairs) generated by the CKSAAP encoding scheme.

**Figure 3:**
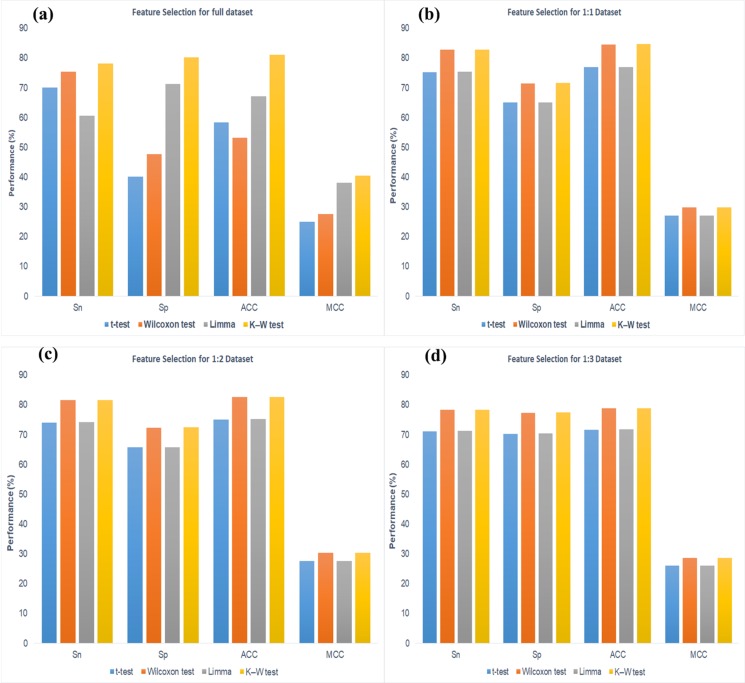
The feature slection performance of different test statistic for (a) Full dataset, (b) 1:1 dataset, (c) 1:2 dataset and (d) 1:3 dataset

**Figure 4:**
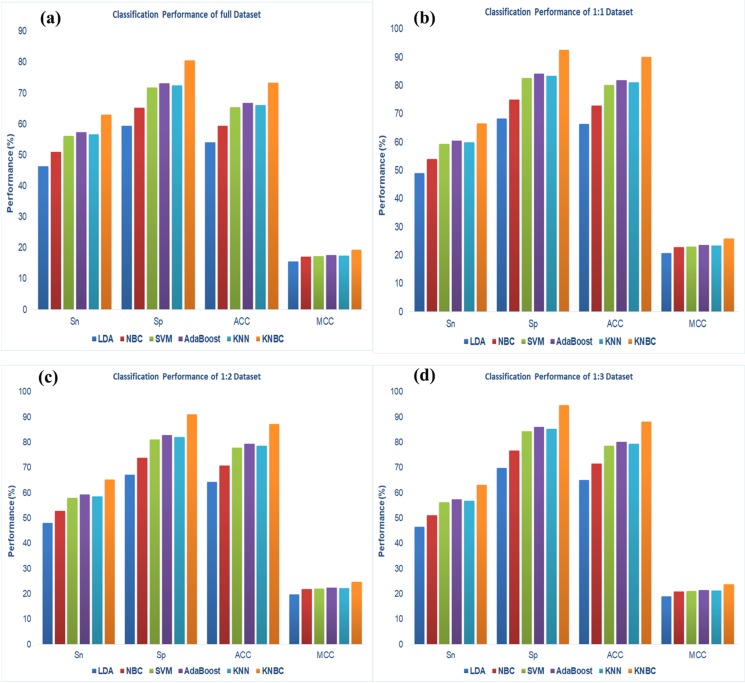
The performance of different classifiers for (a) Full dataset, (b) 1:1 dataset, (c) 1:2 dataset and (d) 1:3 dataset

For the selection of appropriate feature selection method we investigated that the K-W method shown the better performance than others for the full dataset. The Sn (78.21%), Sp (80.23), ACC (81.16%) and MCC (40.43%) are comparative (Figure 4a) with others method like t-test, Wilcoxon and Limma. Similarly we investigate for the balance datasets like as 1:1 (that is number of positive samples (acetylation) and number of negative samples (non-acetylation) are equal), 1:2 (two times negative samples than the positive) and 1:3 (three times negative samples than the positive); the performance of K-W like Sn, Sp, ACC and MCC (Figure 3b-d) are higher than the other methods. This method gives some apparatuses for predicting the acetylation sites; acetylated K sites are importance of these features was also clearly and intuitively characterized of acetylated lysine (K) site prediction, which represents the residue pair spaced by any amino acid.

**Figure 5:**
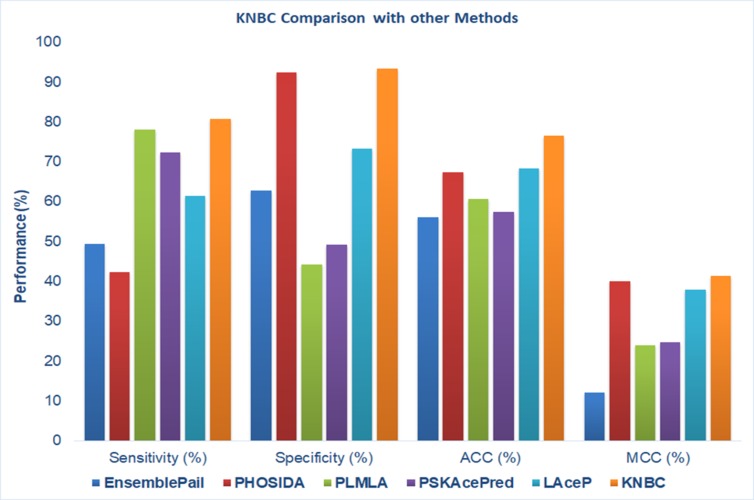
Comparison with other popular methods for acetylation sites prediction

We investigate the selection of appropriate classifiers for classification of acetylation and non-acetylation sites. The performances of KNBC are Sn (63.03%), Sp (80.56%), ACC (73.43%) and MCC (19.40%) comparative with other methods like LDA, NBC, SVM, AdaBoost, KNN. Also we investigate for the balance datasets like as 1:1 (that is number of positive samples (acetylation) and number of negative samples (nonacetylation) are equal), 1:2 (two times negative samples than the positive) and 1:3 (three times negative samples than the positive); the performance of KNBC like Sn, Sp, ACC and MCC (Figure 4a-d) are higher than the other methods. The performance reveals that the KNBC method shows better performance (Figure 5) than the others five available online acetylation site prediction tools. It gives the Sn, Sp, ACC and MCC are 80.71%, 93.39%, 76.43%, and 41.37 respectively. The prediction result shown that the KNBC approach using CKSAAP encoding scheme and top features selected by K-W test with the optimum window size is 47 with center lysine (K) better performance than the others.

## Conclusion

Prediction of protein acetylation sites (PAS) is critical in the understanding of PTM. We describe a prediction model for PAS using Kernel Naive Bayes classifier in this article. The model achieved the accuracy is 76.7% with 81% sensitivity and 94.4% specificity.

## Competing Interests

The authors declare that they have no competing interests.

## Figures and Tables

**Table 1:** AUC between two encoding methods by the proposed method

Encoding Methods	Proposed (AUC)
Binary	0.761
CKSAAP	0.830
